# Longitudinal associations between leisure activities and subjective happiness among middle-aged and older adults people in China: national cohort study

**DOI:** 10.3389/fpubh.2024.1441703

**Published:** 2024-09-13

**Authors:** Chengkai Feng, Zhenguo Shi, Yuge Tian, Chao Ma, Qian Sun

**Affiliations:** School of Physical Education, Shandong University, Jinan, China

**Keywords:** middle-aged and older adults people, leisure activity, physical activity, social activity, subjective happiness

## Abstract

**Background:**

Leisure activities serve as key measures to enhance the subjective happiness of middle-aged and older adults individuals and to positively address the challenges of an aging society; however, the trajectory of changes in their participation in leisure activities and how these changes relate to shifts in subjective happiness have not been adequately explored.

**Methods:**

This study selected data from the China Health and Aging Longitudinal Study (CHARLS), which included a total of 5,190 middle-aged and older adults people. Linear and nonlinear latent growth models, parallel latent growth models and cross-lagged models were constructed to investigate the influence and lagged relationship between the trajectory of changes in the level of participation in leisure activities on the trajectory of changes in the subjective well-being of middle-aged and older adults people.

**Results:**

The initial level of physical activity participation of middle-aged and older adults people significantly predicted the initial level (*β* = 1. 203, *p* < 0.001) and rate of change (*β* = −0.138, *p* = 0.016) of their subjective well-being, and the trajectory of change of middle-aged and older adults people’s physical activity also significantly predicted the rate of change of their subjective well-being (*β* = 0.582, *p* = 0.003). Meanwhile, the initial level of social activity of middle-aged and older adults people also effectively predicted the initial level of their subjective well-being (*β* = 0.048, *p* < 0.001). At the same time, the covariates (gender, age, level of education, marital status, chronic disease) also predicted the initial level and rate of change of leisure activity participation level and subjective well-being. Finally, the cross-lagged model test confirmed the predictive effect of leisure activity participation level on subjective well-being of middle-aged and older adults people.

**Conclusion:**

This study confirms that the level of participation in leisure activities of Chinese middle-aged and older adults people has a significant predictive effect on their subjective happiness and that there is a significant correlation between the trajectory of changes in the level of participation in leisure activities and the trajectory of changes in subjective happiness.

## Background

The subjective happiness of middle-aged and older adults people is a subjective evaluation of their quality of life, which is closely related to their physical and mental health. According to data from the seventh national population census, there are more than 264 million older adults people aged 60 and above in China, accounting for 18.70 per cent of the total population, which is a deepening of the aging of China’s population compared with the 10.3 per cent recorded in 2000 (1). At present, subjective happiness has become an important indicator of the quality of life of the older adults in their later years and the key to positively coping with the aging of the population and has received more and more attention from scholars (2, 3). The subjective happiness of many middle-aged and older adults individuals has been significantly impacted by COVID-19. The suddenness and unpredictability of the pandemic have posed serious threats to mental health and a sense of security globally, leading to a decline in overall well-being (4). The social isolation measures taken by governments to reduce the spread of the virus have led to a lack of social support and a sense of isolation among the majority of older adults people, which also has a serious impact on their subjective sense of well-being (5).

Subjective happiness plays a crucial role in the well-being of middle-aged and older adults people. It is closely intertwined with individual cognition (6). For example, older adults with mild cognitive impairment typically experience lower levels of subjective happiness compared to those with normal cognitive abilities (7). Additionally, subjective happiness is strongly linked to psychological health, with higher levels of happiness predicting greater life satisfaction and more positive emotions (8). Social relationships also play a significant role; social support, for instance, can mitigate the impact of dissatisfaction with one’s image on subjective happiness (9). Furthermore, research suggests that subjective happiness provides strong support for both the physical and mental health of middle-aged and older adults individuals (10). When subjective happiness is low in old age, there will be serious consequences such as decreased physical and mental health, frequent illnesses, and even suicide (11).

Therefore, affected by COVID-19, the lower subjective happiness of middle-aged and older adults people (12, 13) and how to enhance their subjective happiness and thus promote the physical and mental health of middle-aged and older adults people is an important issue that needs to be studied urgently. Activity theory finds that people accomplish specific goals by engaging in activities that promote the development of their psyche and consciousness in the process of engaging in activities and improve subjective happiness (14). At the same time, positive psychology has also found that people’s participation in leisure activities can effectively promote individual psychological health and thus improve their subjective happiness (15). Positive psychology has also found that participation in leisure activities is effective in promoting psychological well-being and subjective happiness. Related studies have also confirmed this idea, for example, in a study of Chilean adolescents, it was found that physical activity can help adolescents to self-regulate their emotions and reduce the impact of negative emotions on themselves, thus improving their subjective happiness (16). The study found that physical activity helped adolescents to self-regulate their emotions and reduce the impact of negative emotions on themselves, thereby increasing subjective happiness. During the COVID-19 epidemic, a review of the impact of contemporaneous physical activity on the mental health of adults confirmed that the epidemic and closure measures led to an increase in sedentary behavior and a decrease in physical activity levels, leading to unhealthy psychological problems such as stress, anxiety, and isolation (17). Health behavior theory suggests that people engage in behaviors, including moderate exercise, socialization, dietary modification, and cessation of bad habits, to improve their physical health, cure illnesses, or increase fitness, thereby enhancing their subjective happiness (18). This theory is supported by relevant studies. For instance, the World Health Organization has suggested that appropriate physical activity can help individuals overcome sedentary behaviors and other unhealthy habits, thereby improving their overall physical health (19). Additionally, in a review study of cancer patients, it was found that those who engaged in regular physical activity experienced better physical health and a higher quality of life compared to those who did not (20). In addition, the theory of need satisfaction suggests that in leisure activities, people can be free from external pressures and free to do what they love, from which they can gain a sense of satisfaction and achievement and satisfy the individual’s need for self-fulfilment. At the same time, the theory also suggests that individual happiness mainly comes from the satisfaction of one’s own needs. Therefore, when people actively participate in leisure activities, their needs for leisure are satisfied, and they will have a sense of happiness (21). The above theories and studies may suggest that leisure activities may improve an individual’s physical health. These theories and studies suggest that leisure activities may have a positive predictive effect on subjective happiness.

However, previous studies usually treat the level of leisure activity participation and subjective happiness as time-invariant variables and simply verify the correlation between the initial levels of the variables (22, 23) and few studies have explored the interaction between leisure activity trajectories and subjective happiness trajectories. Therefore, in this study, linear and nonlinear latent growth models were constructed to examine the initial level and rate of change of the participation in leisure activities (physical and social activities) and subjective happiness of middle-aged and older adults people, using data from four waves of the China Health and Aging Longitudinal Study (CHARLS) spanning a total of 7 years. The parallel latent growth model and the cross-lagged model are also used to investigate the influence of the trajectory of the change in the level of participation in leisure activities on the trajectory of the change in subjective happiness of the middle-aged and the lagged relationship between the level of participation in leisure activities and subjective happiness of the middle-aged and the older adults. We hope to provide theoretical references for improving the subjective happiness of the middle-aged and the older adults and actively coping with population aging.

## Methods

### Sample and data collection

The research data were drawn from the China Health and Retirement Longitudinal Study (CHARLS) database, a national survey that began in 2011. The survey encompassed 150 countylevel units, 450 village-level units, and approximately 10,000 households, covering a total of more than 17,000 individuals. Follow-up surveys were conducted in 2013, 2015, and 2018. The samples for these surveys were selected using a probability sampling method, with probabilities proportional to the population size at each stage of selection—sequentially at the county, village (or neighborhood), household, and individual levels. More details on the CHARLS database can be found in previous publications (24). Four waves of data from this database in 2011 (Wave 1), 2013 (Wave 2), 2015 (Wave 3), and 2018 (Wave 4) were selected for investigation in this study, with a follow-up survey for valid participants in 2011 (Wave 1).

**Figure 1:**
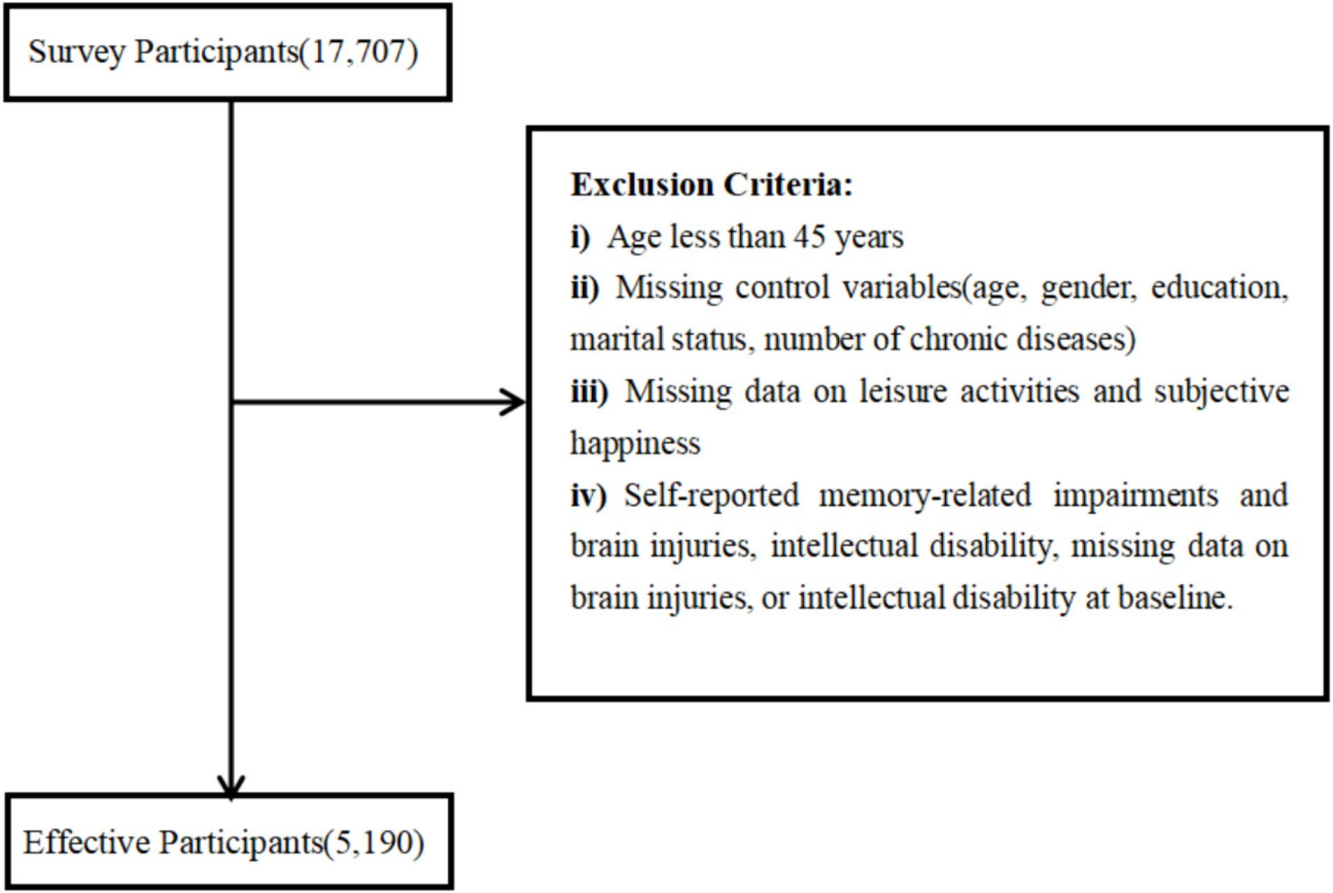
Sampling procedure table.

The purpose of this study was to examine the relationship between leisure activities and subjective happiness among middle-aged and older adults based on a longitudinal study with control of relevant variables. Therefore, the sample exclusion criteria for this study were: age less than 45 years (498 people), missing covariates (age, gender, education, marital status, number of chronic diseases) (466 people), missing participation in leisure activities (4,856 people), missing subjective happiness (4,120 people), self-reported memory-related impairments and brain injuries, intellectual disability, missing data on brain injuries, or intellectual disability at baseline (2,577 people). Ultimately, a total of 5,190 respondents were included after the above operational exclusions (Figure 1).

### Ethical considerations

This study was organized by the National Institute of Development Studies, Peking University, according to the ethical guidelines of the 1975 Declaration of Helsinki, and was approved by the Ethics Committee of Peking University (approval number: IRB00001052-13074). Research involving human participants was approved for review by the Ethics Research Committee of Peking University (approval number: IRB00001052-11015), and subjects gave informed written consent to participate in this study.

### Leisure activity

This study combines the definitions of previous scholars (25) and the characteristics of Chinese middle-aged and older adults people’s leisure activities to classify their leisure activities into physical activities and social activities (26). Based on the characteristics of the CHARLS data, the participation in leisure activities of the middle-aged and older adults was categorized into participation in physical activities (activities related to physical exercise) and participation in social activities (activities related to socializing and making friends).

In addition to this, previous studies have typically measured physical activity participation only through metrics such as exercise intensity, duration, and days of participation (27). Therefore, to reflect the level of physical activity participation of middle-aged and older adults people more comprehensively, the present study, based on combining previous studies and the CHARLS HRQOL scale (28, 29) In this study, we chose to demonstrate the level of physical activity participation of middle-aged and older adults people more comprehensively from the four aspects of PF (physiological function), RP (role-body), BP (body pain), and GH (general health). In this study, PF (Physiological Functioning) mainly measures the level of difficulty in physical activity participation of middle-aged and older adults people; RP (role-body) measures the limitation of physiological cognition on the participation of middle-aged and older adults people in physical activity; BP (Body Pain) is to measure the limitation of physical pain on the participation of middle-aged and older adults people in physical activity; and GH (General Health) is to measure the influence of health status on the participation of middle-aged and older adults people in physical activity. Regarding the measurement of the level of social activity participation, this study referred to previous related studies (30), which measured the total types of social activities that middle-aged and older adults people participated in and the frequency of participation in social activities in the past month. The items measuring physical and social activity were taken from the CHARLS database, and the items did not change over the four waves of data, as shown in Tables 1, 2.

**Table 1:** Measurement items for physical activities.

Variant	Types	Items
Physical activity	PF	Difficulty with running or jogging about 1Km
		Difficulty with walking 1Km
		Difficulty with walking 100 Meters
		Difficulty with getting up from a chair
		Difficulty with climbing several flights of stairs without resting
		Difficulty with stooping, kneeling, or crouching
		Difficulty with reaching or extending your arms
		Difficulty with lifting or carrying weights over 10 Jin
		Difficulty with picking up a small coin
	RP	Difficulty with household chores
		Difficulty with preparing hot meals
		Difficulty with shopping for groceries
		Difficulty with managing money
		Difficulty with taking medications
	BP	Feeling head pain
		Feeling shoulder pain
		Feeling arm pain
		Feeling wrist pain
		Feeling fingers pain
		Feeling chest pain
		Feeling stomach pain
		Feeling back pain
		Feeling waist pain
		Feeling buttocks pain
		Feeling leg pain
		Feeling knees pain
		Feeling ankle pain
		Feeling toes pain
		Feeling neck pain
	GH	Self comment of your health

PF is physiological function; RP is role-body; BP is body pain; GH is general health.

**Table 2:** Measurement items for social activities.

Variant	Items
Social activity	How often do activities of interacted with friends
	How often do activities of played Ma-jong/Cards/Chess or went to community club
	How often do activities of provided help to people who lived apart
	How often do activities of went to club
	How often do activities of took part in a community-related organization
	How often do activities of voluntary or charity
	How often do activities of cared for a sick or disabled adult who lived apart
	How often do activities of attended an educational or training course
	How often do activities of stock investment
	How often do activities of used the internet
	How often do activities of other
	How often do activities of none of these

In this study, partial data of the two types of activity measures were assigned in reverse to ensure that higher scores indicate more active participation in activities. Meanwhile, according to the scoring rules of the SF-36 scale, physical activities were divided into 4 dimensions, and each question item was assigned a score according to a scale of 1–4, while social activities were assigned a score of 1 for participation in an activity. Raw scores for the degree of participation in the 2 types of activities in their respective components were computed, and scores for the 2 types of activities were converted to a range of 0–100 for analyses (28, 29). The total score for both physical activity and social activity was 100. Finally, the Cronbach’s α coefficients for the physical activity participation level and the social activity participation level were 0.764 and 0.655, respectively, and the Cronbach’s α coefficients for the 2 activity participation levels were greater than 0.6, indicating that this scoring method has a certain degree of reliability.

### Subjective happiness

Subjective happiness in this study was measured using the CHARLS Depression Scale (CES-D), which is a simplified version of the depression scale developed by Radolff of the National Institute of Mental Health in the U.S. Relevant studies have demonstrated that the CES-D can also be applied to middle-aged and older adults people in China (31). The CES-D consists of 10 questions that measure the subject’s worry, energy, fear, sleep quality, happiness, hope, and other dimensions, and is effective in measuring subjective happiness (32). In this study, the relevant data were reverse-assigned to ensure that the higher the subject’s score, the higher the subjective happiness. Each question was scored using a four-level scale, with each question item maintaining a score ranging from 0–3, and the total score ranging from 0–30, and the Cronbach’s alpha coefficient for subjective happiness was 0.806 greater than 0.6, which indicates that this type of scoring method has a high degree of reliability.

### Control variable

The Control Variable in this study mainly included age, gender, education level, marital status, and number of chronic diseases of the subjects (Table 3). All covariates were measured during the first wave (WAVE 1) of the survey and were included in the study as covariates of the initial level and rate of change in the level of participation in leisure activities (physical and social activities) and subjective happiness among middle-aged and older adults. Gender was categorized as “male” and “female”; age was categorized as “45–50 years” “51–55 years” “56–60 years” “61–65 years” and “Over 65 years”; education level was categorized as “Elementary school and below” “junior high school” “High school, vocational high school” “specialized training school” “Undergraduate and above.” Marital status includes “married” and “not married.” Chronic diseases are self-reported and labeled 0–14 depending on the number of chronic diseases.

**Table 3:** Demographic information.

Variables	Item	Number	Percentage (%)
Gender	Male	2,591	49.9
	Female	2,599	50.1
Age	45–50 years	1,366	26.3
	51–55 years	1,001	19.2
	56–60 years	1,240	23.8
	61–65 years	911	17.5
	Over 65 years	672	12.9
Level of education	Elementary school and below	3,201	61.7
	Junior high school	1,297	25.0
	High school, vocational high school	603	11.6
	specialized training school	73	1.4
	Undergraduate and above	16	0.3
Marital status	Married (living together; not living together)	4,801	92.5
	Not in marriage (divorced; widowed; unmarried)	389	7.5
Chronic disease	0 species	1746	33.6
	1 species	1,587	30.5
	2 species	975	18.8
	3 species	494	9.5
	4 species	236	4.5
	5 species	105	2.0
	6 species	38	0.7
	7 species	9	0.2

## Results

### Common method bias testing

To avoid the influence of a single sample on the increase or decrease of correlation between dimensions and to ensure the scientific validity of the study. Therefore, this study conducted a Harman’s one-way test on the data, such methods are usually used as a test of the extent of common method bias, the results of the test showed that the first factor explains 34.7% of the variance, which is less than the desirable value of 40%, so there is no common method bias in this study (33).

### Statistical description

**Figure 2:**
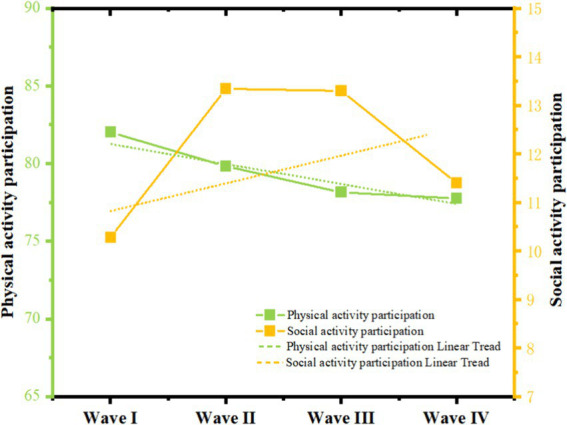
Trajectory of participation levels in leisure activities (physical and social activities).

**Figure 3:**
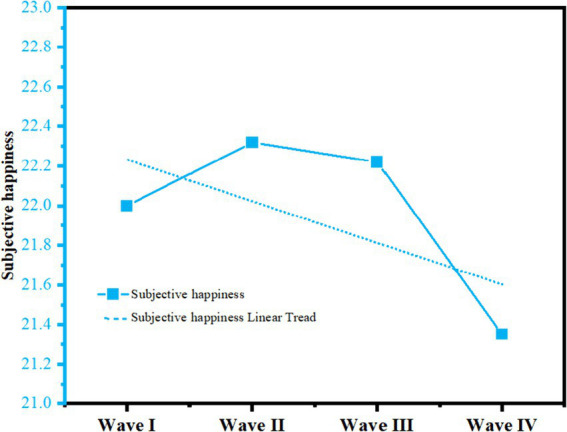
Trajectory of subjective happiness.

Statistical descriptions of the 3 categories of variables at baseline are shown in Table 4, and the results indicate that from 2011 to 2018, the level of participation in social activities, the level of participation in physical activities, and the subjective happiness of middle-aged and older adults have ranged from 10.28 (13.44) to 11.40 (15.13), and from 82.01 (11.86) to 77.76 (14.97) versus 22.00 (6.10), respectively to 21.35 (6.55), Figures 2, 3 below show more clearly the scores and trends of the three types of variables. Table 5 shows the correlation between social activity participation, physical activity participation, and subjective happiness in the four surveys, and as expected, the results show a significant positive correlation between social activity participation, physical activity participation, and subjective happiness from 2011 to 2018.

**Table 4:** Descriptive analysis of variables.

Variable	Wave I, mean (SD)	Wave II, mean (SD)	Wave III, mean (SD)	Wave IV, mean (SD)
SAP	10.28 (13.44)	13.34 (15.59)	13.30 (16.59)	11.40 (15.13)
PAP	82.01 (11.86)	79.83 (10.94)	78.15 (13.63)	77.76 (14.97)
HAP	22.00 (6.10)	22.32 (5.69)	22.22 (6.26)	21.35 (6.55)

SAP is social activities; PAP is physical activities; HAP is subjective happiness.

**Table 5:** Matrix of correlation coefficients.

Variables	T1_SAP	T2_SAP	T3_SAP	T4_SAP	T1_PAP	T2_PAP	T3_PAP	T4_PAP	T1_HAP	T2_HAP	T3_HAP	T4_HAP
T1_SAP	1											
T2_SAP	0.311^**^	1										
T3_SAP	0.313^**^	0.364^**^	1									
T4_SAP	0.259^**^	0.312^**^	0.368^**^	1								
T1_PAP	0.016	0.042^**^	0.039^**^	0.042^**^	1							
T2_PAP	0.075^**^	0.090^**^	0.109^**^	0.096^**^	0.260^**^	1						
T3_PAP	0.070^**^	0.101^**^	0.090^**^	0.089^**^	0.263^**^	0.602^**^	1					
T4_PAP	0.073^**^	0.096^**^	0.116^**^	0.099^**^	0.286^**^	0.594^**^	0.663^**^	1				
T1_HAP	0.093^**^	0.114^**^	0.097^**^	0.073^**^	0.278^**^	0.398^**^	0.405^**^	0.413^**^	1			
T2_HAP	0.083^**^	0.091^**^	0.080^**^	0.083^**^	0.220^**^	0.487^**^	0.442^**^	0.430^**^	0.522^**^	1		
T3_HAP	0.064^**^	0.088^**^	0.091^**^	0.065^**^	0.217^**^	0.412^**^	0.537^**^	0.468^**^	0.497^**^	0.570^**^	1	
T4_HAP	0.085^**^	0.097^**^	0.100^**^	0.075^**^	0.201^**^	0.376^**^	0.421^**^	0.518^**^	0.443^**^	0.501^**^	0.542^**^	1

T1 is the first wave; T2 is the second wave; T3 is the third wave; T4 is the fourth wave. SAP is a social activity; PAP is Physical activity; HAP is subjective happiness. ^**^p < 0.01.

### Trajectories of physical and social activity participation among middle-aged and older adults

To explore the relationship between subjective happiness and the participation level of leisure activities (physical activities and social activities) among middle-aged and older adults people, we used unconditional linear and nonlinear models to assess the initial subjective happiness, physical activity participation level, social activity participation level of middle-aged and older adults people, as well as the trajectory of their changes over time, and the relevant results and model fit indices, are shown in Table 6.

**Table 6:** Unconditional linear and nonlinear latent variable growth model fit indices and model results.

Model	*χ*2	df	RMSEA	CFI	TLI	SRMR	Estimate			Variance		
							Intercept	Slope	Quadratic	Intercept	Slope	Quadratic
unconditional linear model												
PAP	273.008	5	0.102	0.958	0.95	0.135	81.132^***^	−0.538^***^		33.032^***^	1.678^***^	
SAP	301.894	5	0.107	0.885	0.862	0.05	11.566^***^	0.071^*^		73.618^***^	1.712^***^	
HAP	114.402	5	0.065	0.983	0.98	0.025	22.330^***^	−0.102***		18.767^***^	0.137***	
unconditional nonlinear model												
PAP	2.343	1	0.016	1	0.999	0.004	82.094^***^	−1.406^***^	0.112^***^	22.478^***^	8.905^***^	0.111^***^
SAP	10.003	1	0.042	0.997	0.979	0.009	10.400^***^	1.724^***^	−0.227^***^	47.142^***^	2.194	0.076
HAP	0.013	1	0	1	1.001	0	22.005^***^	0.256^***^	−0.050^***^	15.914^***^	−0.1	0.013^*^

PAP is physical activity; SAP is a social activity; HAP is subjective happiness. ^*^p < 0.05; ^***^p < 0.001.

As shown in Table 6, the fit of the nonlinear model for the potential growth of physical activity participation levels for middle-aged and older adults people was better than the linear model, so the nonlinear model for physical activity participation was chosen for the study. The slope of the quadratic variance of the level of physical activity participation was 0.111 (*p* < 0.001), the initial level (intercept) of physical activity participation was 82.094 (*p* < 0.001), the level of physical activity participation decreased year by year in the 4-wave test (slope = −1.406, *p* < 0.001), and the rate of decline increased year by year (slope = 0.112, *p* < 0.001), indicating that physical activity participation level has a non-linear decreasing trend in the 4-wave test. Meanwhile, the intercept variance and slope variance of physical activity participation levels were 22.478 (*p* < 0.001) & 8.905 (*p* < 0.001), respectively, indicating a significant difference in the rate of decline among individuals.

As shown in Table 6, for the level of social activity participation of middle-aged and older adults people, the potential growth nonlinear model fit better than the linear model, so the quadratic term model of the level of social activity participation was chosen to be analyzed in this study. The initial level (intercept) of social activity participation was 10.4 (*p* < 0.001), and the level of social activity participation increased year by year in the 4-wave test (slope = 1.724, *p* < 0.001), and the growth rate decreased year by year (slope = −0.227, *p* < 0.001), indicating that the level of social activity participation showed a nonlinear growth trend in the 4-wave test. The intercept variance and slope variance of the social activity participation level were 47.142 (*p* < 0.001) & 2.194, respectively.

### Subjective happiness trajectories in middle-aged and older adults

As shown in Table 6, for the subjective happiness of middle-aged and older adults people, although the goodness of fit of both linear and nonlinear models of their potential growth is acceptable, the quadratic slope variance and quadratic variance of the nonlinear model are not significant. Therefore, the linear model was chosen for analysis in this study. The results showed that the intercept variance and slope variance of subjective happiness were 18.767 (*p* < 0.001) and 0.137 (*p* < 0.001), respectively, which indicated that there were significant differences in the initial level and rate of decline of subjective happiness among middle-aged and older adults individuals. Meanwhile, the slope of subjective happiness was −0.102 (*p* < 0.001), indicating that subjective happiness showed a linear decreasing trend from year to year in the 4 waves of the test.

### Parallel LGCM

**Figure 4:**
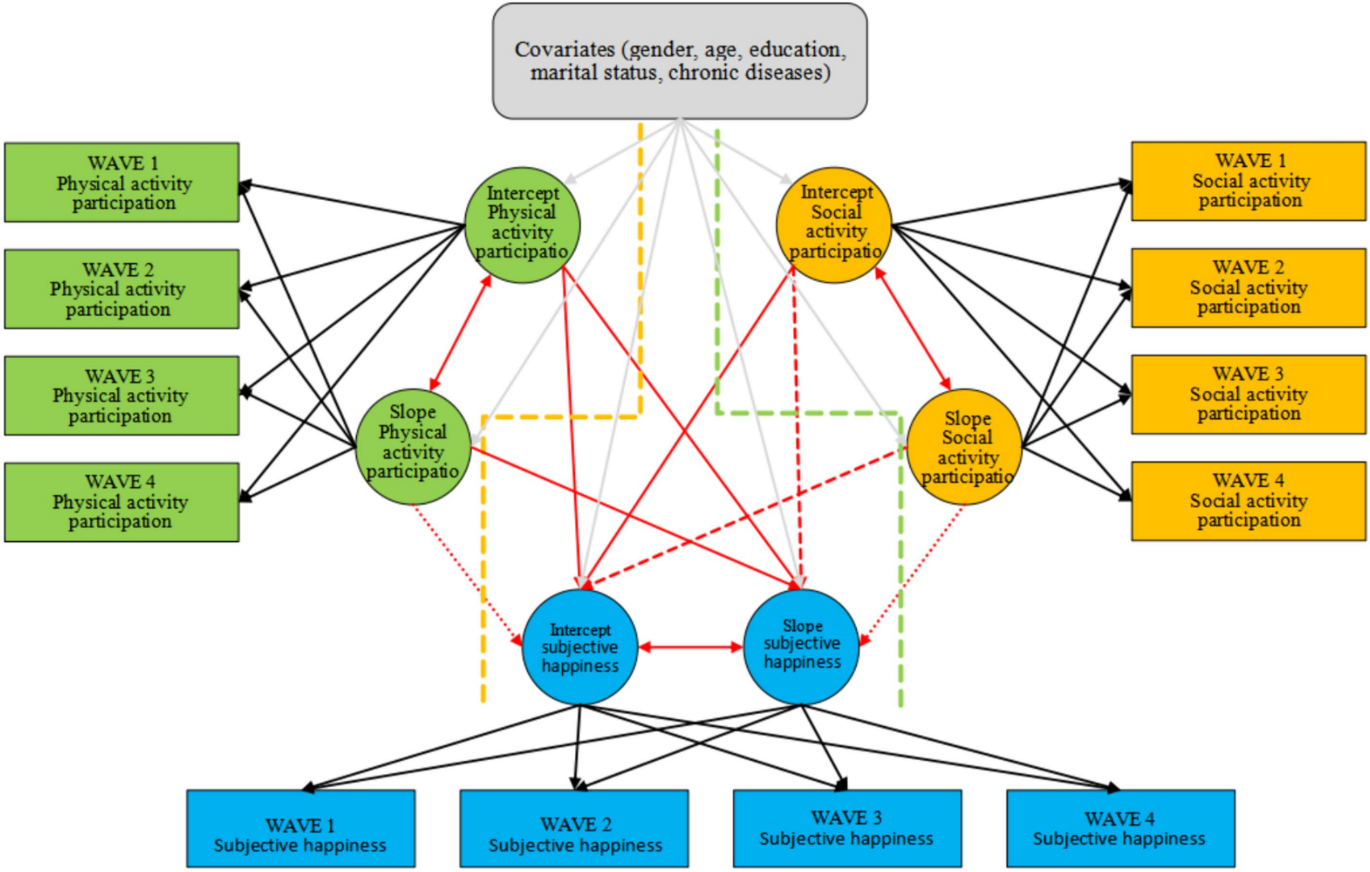
Parallel latent growth model of leisure activity participation level and subjective happiness of middle-aged and older adults people. Valid and invalid paths are indicated by solid and dashed lines, respectively.

In order to more accurately study the relationship between subjective well-being and leisure activities of middle-aged and older adults people, as well as the trends of both over time, we used a parallel LGCM in this section (Figure 4). The parallel LGCM focuses on the relationship between the intercept and slope of physical activity participation and social activity participation on the intercept and slope of subjective happiness. Unstandardized coefficients were reported inside the parallel latent growth model due to the 0.9+ correlation between the intercept for physical activity and the intercept for well-being in the parallel latent growth model. As shown in Table 7, the fit index of the parallel latent growth model constructed by the level of participation in leisure activities (physical activities and social activities) and subjective happiness of middle-aged and older adults people is in the acceptable range.

**Table 7:** Parallel latent growth model fit indices.

Fit	*χ*2	df	RMSEA	CFI	TLI	SRMR
Model	1232.241	81	0.052	0.946	0.916	0.047

In Figure 4 effective and ineffective routes are represented by solid and dashed lines, respectively. The results of the study are shown in Table 8, first from the initial level of subjective happiness of middle-aged and older adults people. There is a positive effect of the initial level of physical activity participation on the initial level of subjective happiness (*β* = 1.203, *p* < 0.001), indicating that the higher the initial level of physical activity of middle-aged and older adults people, the higher the subjective happiness. However, the rate of decline in the level of participation in physical activity was not correlated with the initial level of subjective happiness (*β* = −1.799, *p* = 0.103). Meanwhile, the initial level of social activities positively affected the initial level of subjective happiness (*β* = 0.048, *p* < 0.001), indicating that the higher the initial level of social activities, the stronger the subjective happiness of middle-aged and older adults people. However, the rate of increase in the level of participation in social activities was not correlated with the initial level of subjective happiness (*β* = −0.195, *p* = 0.151).

**Table 8:** Parallel latent growth model path coefficients.

Path	Estimate	S.E.	*p*
I_pap_ → I_hap_	1.203	0.331	0
S_pap_ → I_hap_	−1.799	1.104	0.103
I_sap_ → I_hap_	0.048	0.011	0
S_sap_ → I_hap_	−0.195	0.136	0.151
I_pap_ → S_hap_	−0.138	0.057	0.016
S_pap_ → S_hap_	0.582	0.194	0.003
I_sap_ → S_hap_	−0.003	0.002	0.233
S_sap_ → S_hap_	0.007	0.025	0.776

I is the intercept; S is the slope. pap is physical activity; sap is a social activity; hap is subjective happiness.

As shown in Table 8, on the other hand, from the subjective happiness decline rate of middle-aged and older adults people, the initial level of physical activity participation negatively predicts the decline rate of subjective happiness (*β* = −0.138, *p* = 0.016), indicating that the higher the initial level of physical activity participation, the slower the decline rate of subjective happiness. Meanwhile, the decline rate of physical activity participation has a positive effect on the decline rate of subjective happiness (*β* = 0.582, *p* = 0.003), indicating that the higher the decline rate of middle-aged and older adults people’s physical activity participation, the faster the decline rate of their subjective happiness. In contrast, both the initial level (*β* = −0.003, *p* = 0.233) and the rate of increase (*β* = 0.007, *p* = 0.776) of middle-aged and older adults people’s participation in social activities were not correlated with the rate of decline in subjective happiness.

According to Table 9, the number of chronic diseases has a significant effect on the initial level of subjective happiness of middle-aged and older adults people (*β* = 1.617, *p* < 0.05), indicating that middle-aged and older adults people with a lower number of chronic diseases have a higher initial level of subjective happiness. There is a significant effect of education level and age on subjective happiness of middle-aged and older adults people (*β* = 0.164, *p* < 0.05; *β* = 0.019, *p* < 0.05), indicating that the higher the education level and the older the middle-aged and older adults people, the faster the rate of decline of subjective happiness. Middle-aged and older adults people’s gender, education level, age, and chronic diseases all had a significant effect on their initial level of physical activity (*β* = −1.897, *p* < 0.001; *β* = 1.486, *p* < 0.001; *β* = 0.039, *p* < 0.001; *β* = −2.495, *p* < 0.001), indicating that the higher the level of education of middle-aged and older adults people, the higher the level of education of middle-aged and older adults people, and the higher the age of middle-aged and older adults, and the lower the number of chronic diseases, the higher the initial level of physical activity participation. The gender, education level, age and chronic diseases of middle-aged and older adults people all also have a significant effect on the rate of decline in physical activity (*β* = −0.415, *p* < 0.001; *β* = 0.133, *p* < 0.001; *β* = −0.020, *p* < 0.001; *β* = −0.165, *p* < 0.001), indicating that middle-aged and older adults females, middle-aged and older adults people with less education, older middle-aged and older adults people, and the lower number of chronic diseases older middle-aged and older adults, and middle-aged and older adults with a higher number of chronic diseases have a slower rate of decline in physical activity participation. The significant effects of gender, education level, and age on the initial level of participation in social activities (*β* = −1.575, *p* < 0.001; *β* = −2.928, *p* < 0.001; *β* = −0.155, *p* < 0.001) indicate that middle-aged and older adults males, middle-aged and older adults people with higher education levels, and middle-aged and older adults people with lower ages have higher initial levels of participation in social activities. The gender and education level of middle-aged and older adults people have a significant effect on the growth rate of their social activities (*β* = 0.219, *p* < 0.001; *β* = 0.247, *p* < 0.001), indicating that middle-aged and older adults women and middle-aged and older adults people with higher education levels have a faster growth rate of participation in social activities.

**Table 9:** Effect of covariates on leisure activities (physical activities, social activities) and subjective happiness of middle-aged and older adults people.

Variables	I-hap	S-hap	I-pap	S-pap	I-sap	S-sap
Gender	0.441	−0.104	−1.897^***^	−0.415^***^	−1.575^***^	0.219^***^
Level of education	−0.624	0.164^*^	1.486^***^	0.133^***^	2.928^***^	0.247^***^
Age	−0.055	0.019^*^	0.039^***^	−0.020^***^	−0.155^***^	−0.004
Marital status	−1.351	0.048	−0.402	−0.018	0.589	0.016
Chronic disease	1.617^*^	−0.194	−2.495^***^	−0.165^***^	0.219	0.005

I is the intercept; S is the slope. pap is physical activity; sap is a social activity; hap is subjective happiness. ^*^p < 0.05; ^***^p < 0.001.

### Cross-lag model

Cross-lagged modeling is the best way to test the “single” effect between variables (34). In the present study, the cross-lagged model was used to further examine the relationship between the level of participation in leisure activities (physical and social activities) and the subjective happiness of middle-aged and older adults people over time and to verify the robustness of the predictive effect of the level of participation in leisure activities (physical and social activities) on the subjective happiness of middle-aged and older adults people. However, it is worth noting that latent variable cross-lagged models are prone to saturated models and usually do not require excessive attention to model fit indices; therefore, the fit indices of cross-lagged models are not reported in this study (35).

**Figure 5:**
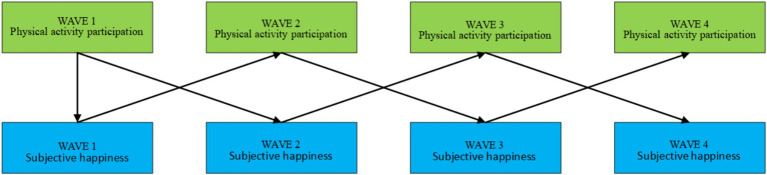
Cross-lagged model of physical activity participation level and subjective happiness of middle-aged and older adults people.

As shown in Figure 5, it was found that the level of physical activity participation of middle-aged and older adults people in the first wave significantly and positively predicted the subjective happiness of middle-aged and older adults people in the second wave (*β* = 0.055, *p* < 0.001) through the study of the results of the 4 waves of survey. The level of participation in physical activity in the second wave of middle-aged and older adults people significantly and positively predicted the subjective happiness of middle-aged and older adults people in the third wave (*β* = 0.158, *p* < 0.001). The third wave of the physical activity participation level of middle-aged and older adults people significantly positively predicted the fourth wave of subjective happiness (*β* = 0.160, *p* < 0.001). Meanwhile, the subjective happiness of middle-aged and older adults people in the previous wave also had a significant positive predictive effect on their physical activity participation, and the results are shown in Table 10.

**Table 10:** Regression path coefficients for cross-lagged models.

Regression path	Wave I to Wave II	Wave II to Wave III	Wave III to Wave IV
Physical activity participation and subjective happiness			
PAP → HAP	0.055^***^ (0.013)	0.158^***^ (0.013)	0.160^***^ (0.014)
HAP → PAP	0.288^***^ (0.013)	0.175^***^ (0.012)	0.148^***^ (0.012)
HAP → HAP	0.469^***^ (0.012)	0.450^***^ (0.012)	0.427^***^ (0.013)
PAP → PAP	0.094^***^ (0.013)	0.450^***^ (0.012)	0.515^***^ (0.011)
Social activity participation and subjective happiness			
SAP→HAP	0.031^*^ (0.012)	0.020 (0.012)	0.035^*^ (0.012)
HAP → SAP	0.061^***^ (0.014)	0.031^*^ (0.013)	0.017 (0.013)
HAP → HAP	0.477^***^ (0.011)	0.534^***^ (0.010)	0.500^***^ (0.011)
SAP→SAP	0.272^***^ (0.013)	0.323^***^ (0.012)	0.327^***^ (0.012)

PAP is physical activity; SAP is a social activity; HAP is subjective happiness. ^*^p < 0.05; ^***^p < 0.001.

**Figure 6:**
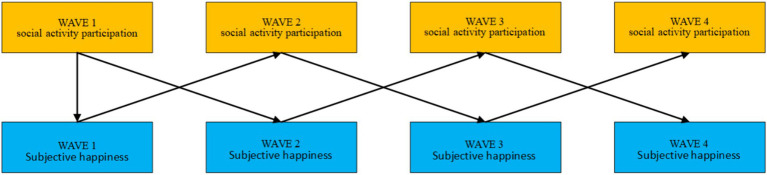
Cross-lagged model of social activity participation level and subjective happiness of middle-aged and older adults people.

As shown in Figure 6, the cross-lagged model of middle-aged and older adults people’s social activity participation level and subjective happiness demonstrated similar results. In the first wave of middle-aged and older adults In the second wave, the situation of middle-aged and older adults social activity participation level has a significant positive predictive effect on the subjective happiness of middle-aged and older adults in the second wave (*β* = 0.031, *p* < 0.05). There was no significant correlation between the level of participation in social activities of middle-aged and older adults people in the second wave and their subjective happiness in the third wave (*β* = 0.020, *p* = 0.092). There was a significant positive prediction of the third wave of physical activity participation of middle-aged and older adults people on their fourth wave of subjective happiness (*β* = 0.035, *p* < 0.05). At the same time, older adults’ subjective happiness also had a significant positive effect on their subsequent level of social activity participation, and the results are shown in Table 10.

## Discussion

Based on data from four CHARLS surveys, this study examined the predictive role of trajectories of change in the participation levels of leisure activities (physical and social activities) of older adults on the trajectories of change in their subjective well-being. First, this study constructed unconditional linear and nonlinear models to assess the initial subjective well-being, initial physical activity participation level, initial social activity participation level, and their rates of change over time among middle-aged and older adults. The results of the study found that the physical activity participation levels of middle-aged and older adults people in China showed a decreasing trend, indicating that the physical activity participation levels of middle-aged and older adults people gradually decrease with age. This finding is supported by related studies, for example, that frailty and symptoms of skeletal muscle loss increase with age in older adults (36). In addition, cognitive function declines in older adults with age (37), and these may be important factors contributing to declining levels of physical activity participation in middle-aged and older adults.

However, the results of the study found that the level of participation in social activities of middle-aged and older adults people showed an increasing trend, but it was mainly concentrated in wave 1 to wave 2, which may be due to the fact that the middle-aged and older adults people selected for this study were ≥ 45 years old, when some of them were in the stage of retirement (the legal retirement age for Chinese citizens is 60 years old), with more leisure time, and in relatively good physical condition, so the level of participation in social activities of the middle-aged and older adults people increased significantly in this stage. Therefore, the level of participation in social activities of the middle-aged and the older adults in this stage rises significantly. During the third and fourth waves, the level of participation in social activities of the middle-aged and the older adults tends to decline. At this point, as they grow older, middle-aged and older adults people are affected by their physical conditions and illnesses, and their participation in social activities decreases. This is also consistent with previous research, such as the fact that the physical and mental health of middle-aged and older adults people is affected by their age, which affects the level of participation in daily social activities (38). At the same time, it is worth mentioning that with the popularization and development of information technology, digital networks have become a key channel for social activities, but as middle-aged and older adults people grow older, the possibility of their participation in social activities through digital networks is gradually isolated, which is also an important factor affecting the level of their participation in social activities (39).

The findings found a downward trend in subjective well-being among middle-aged and older Chinese adults, suggesting that the trajectory of subjective well-being is influenced by age. Related studies have also confirmed this result, such as in a Chinese national cohort study, which found that increasing age amplified depression perception status, and that increased depression perception decreased subjective well-being in middle-aged and older adults (3). In addition, a study in Portugal found that self-esteem progressively decreases with age, while stress and depression progressively increase, leading to a decrease in subjective well-being and life satisfaction (40).

Secondly, according to the results of the LGCM model, it was found that the higher the initial level of participation in leisure activities (sports and social activities) of middle-aged and older adults people, the higher the initial level of their subjective well-being. At the same time, the faster the level of participation in leisure activities of middle-aged and older adults people decreases, the faster their subjective well-being decreases. This may be due to the fact that leisure activities can help middle-aged and older adults people to alleviate adverse emotions such as anxiety and depression, thus increasing their subjective well-being (41). In addition, long-term participation in leisure activities can also help middle-aged and older adults to establish social networks, enhance their social interactions, and reduce their loneliness, thereby increasing their subjective well-being (42). This study also found that the subjective well-being of middle-aged and older adults also affects the level of participation in leisure activities to some extent. This may be due to the fact that well-being contributes to the improvement of subjective physical and mental health of older adults, which in turn promotes the level of participation in physical and social activities (43).

Finally, this study conducted a cross-lagged analysis of the level of participation in leisure activities and the subjective happiness of middle-aged and older adults people to test the “single” effect between the variables and to explore the time-series relationship between the level of participation in leisure activities and the subjective happiness of middle-aged and older adults people. The results of the study show that the level of participation in leisure activities (physical and social activities) of middle-aged and older adults people can positively predict their subjective happiness, and the results of the study also show that the subjective happiness of middle-aged and older adults people can also predict the level of participation in leisure activities (physical and social activities) in the following period. The results of the study further confirmed the correlation between the participation level of middle-aged and older adults people in leisure activities and their subjective happiness (44).

### Limitations

Although the data selected for this paper came from the subjective responses of middle-aged and older adults people, and data that may affect the accuracy of the results of the study, such as cognitive impairment, intellectual disability, and memory problems, were screened out, the subjective responses may have a certain adverse impact on the results of the study.

The focus of the study was to analyze the impact of the level of participation in leisure activities of middle-aged and older adults people on their subjective sense of well-being, and although the study also showed that the subjective sense of well-being of middle-aged and older adults people has an impact on their level of participation in leisure activities, it failed to explain this impact in depth. Future research should also focus on the impact of subjective happiness on the level of participation in leisure activities.

## Conclusion

The results of the study show that there is a bidirectional influence relationship between leisure activities and subjective happiness of middle-aged and older adults people. Leisure activities not only affect the initial level of subjective happiness of middle-aged and older adults people but also influence the trajectory of subjective happiness. Although both physical and social activities have a positive impact on the subjective happiness of middle-aged and older adults people, physical activities have a more direct and significant impact on the subjective happiness of middle-aged and older adults people by improving their physical health and providing them with positive emotions, whereas the impact of social activities on the subjective happiness of middle-aged and older adults people is mainly generated through social interactions and emotional support, which is not as significant as the impact of physical activities.

Therefore, further attention should be paid to the subjective happiness of middle-aged and older adults with lower levels of participation in leisure activities in future research. These findings can help managers actively organize physical activities for middle-aged and older adults people and encourage them to actively participate in social activities, thus effectively improving their subjective happiness.

## Data Availability

The original contributions presented in the study are included in the article/supplementary material, further inquiries can be directed to the corresponding author.
